# Coevolution of Axon Guidance Molecule Slit and Its Receptor Robo

**DOI:** 10.1371/journal.pone.0094970

**Published:** 2014-05-06

**Authors:** Qi Yu, Xiao-Tong Li, Xiao Zhao, Xun-Li Liu, Kazuho Ikeo, Takashi Gojobori, Qing-Xin Liu

**Affiliations:** 1 Laboratory of Developmental Genetics, Shandong Agricultural University, Tai'an, Shandong, China; 2 Center for Information Biology and DNA Data Bank of Japan, National Institute of Genetics, Mishima, Shizuoka, Japan; Vlaams Instituut voor Biotechnologie and Katholieke Universiteit Leuven, Belgium

## Abstract

Coevolution is important for the maintenance of the interaction between a ligand and its receptor during evolution. The interaction between axon guidance molecule Slit and its receptor Robo is critical for the axon repulsion in neural tissues, which is evolutionarily conserved from planarians to humans. However, the mechanism of coevolution between Slit and Robo remains unclear. In this study, we found that coordinated amino acid changes took place at interacting sites of Slit and Robo by comparing the amino acids at these sites among different organisms. In addition, the high level correlation between evolutionary rate of Slit and Robo was identified in vertebrates. Furthermore, the sites under positive selection of *slit* and *robo* were detected in the same lineage such as mosquito and teleost. Overall, our results provide evidence for the coevolution between Slit and Robo.

## Introduction

Molecular coevolution is the reciprocal change in interacting loci during evolution [1]. Coevolution of interacting proteins is important for the maintenance of their interaction and molecular function. The molecular mechanisms that give rise to protein coevolution are complicated. Recently, many computational methods have been developed for the detection of molecular coevolution [2]. The similarity of phylogenetic trees was used to identify the coevolutionary relationships between a large number of ligand-receptor pairs, such as the insulin-insulin receptor and the chemokine-chemokine receptor [3], [4]. Coordinated amino acid changes were found in the hemopoietic ligands and their receptors and bursicon ligand–receptor system [5], [6]. Knowledge of mechanism underlying coevolution between ligand and receptor is essential for understanding the evolutionary process of complex biological systems. The Slit and its receptor Robo are important for axon guidance, neuronal cell migration, neuronal morphological differentiation, tumor metastasis, angiogenesis and heart morphogenesis [7]–[12]. During nervous system development, the guidance cue Slit protein interacted with its receptor Robo to direct the axons to their targets [13], [14]. The interaction of Slit and Robo was confirmed in the planarian in which central nervous system has appeared [15]. Then, the interaction between Slit and Robo was evolutionarily conserved from planarians to humans [13], [16], [17]. However, the mechanism of coevolution between Slit and Robo is unclear.

In this study, we performed evolutionary analysis to search for evidence of coevolution between Slit and Robo. We showed that the interacting amino acids of Slit and Robo exhibited coordinated changes during evolution. We also obtained the high Pearson's correlation coefficient between phylogenetic distance matrices of Slit and Robo. The sites under positive selection of *slit* and *robo* were identified in the same species.

## Materials and Methods

### Data Retrieval and Identification

Sequences of *slit* and *robo* were identified using BLAST searches against the National Center for Biotechnology Information (http://www.ncbi.nlm.nih.gov) and the Ensembl Genome Browser (http://www.ensembl.org). Accession numbers and species were compiled in Tables S1, S2, S3, and S4. Two genes generated by a duplication of *robo1* were termed *robo1a* and *robo1b* in mosquitoes. Two co-orthologous copies of *robo1*, *robo2* and *robo3* were termed *robo1a*, *robo1b*, *robo2a*, *robo2b*, *robo3a* and *robo3b* in teleosts. Two *slit1* orthologues were named *slit1a* and *slit1b* according to the nomenclature in zebrafish [18].

### Analysis for Changes of Interacting Sites

Protein-coding sequences of Slit and Robo (Table S1) were aligned by the MUSCLE program in MEGA 5.05 [19]. Interacting amino acids of *slit* and *robo* according to the five binding sites in human [20] were listed in Table S5. Phylogenetic distribution of interacting amino acids between Slit and Robo is based on recent studies [21].

### Regressions of Protein Distances for Slit Ligands and Robo Receptors

The MirrorTree approach was used to assess the degree of correlated evolution between Slit ligands and Robo receptors. The multiple sequence alignments of Slit1, Slit2, Slit3, Robo1, Robo2 and Robo3 orthologous proteins from 23 vertebrate species (Table S2) were performed by the MUSCLE program. Distances matrices for the orthologues were constructed from the multiple sequence alignments by MEGA 5.05 with pairwise deletion and Poisson correction for amino acids substitution. We calculated the Pearson's correlation coefficient between the distance matrices using the statistics software SPSS. We chose glyceraldehyde 3-phosphate dehydrogenase (Gapdh) as the negative control. The significant differences between correlation values of Slit-Robo pairs and controls were calculated according to Preacher [22].

### Detection of Selective Pressures

The neighbor joining (NJ) trees of Slit and Robo were constructed with MEGA 5.05, and the topologies were used for the following selective pressure analysis. We applied branch-site model (Model A) in the PAML software v.4.4 to test positive selection acting on individual sites along specific branches of the tree [23], [24]. In the branch-site model A, referred to as alternative hypothesis H_1_, branches in the tree are divided a priori into foreground and background categories, and only foreground lineages may have experienced positive selection. This model assumes four classes of sites. Site class 0 and 1 include codons that are conserved (0<ω<1) and evolving neutrally (ω = 1) throughout the tree, and site classes 2a and 2b include codons that are conserved or neutral on the background branches, but become under positive selection on the foreground branches with ω_2_>1. The null hypothesis Model A H_0_ is the branch-site model A with ω_2_ = 1 fixed. The sites under positive selection of *slit* and *robo* were identified by comparing the two models by likelihood ratio test (LRT).

## Results

### Coordinated Changes of Interacting Amino Acids between Slit and Robo

**Figure 1:**
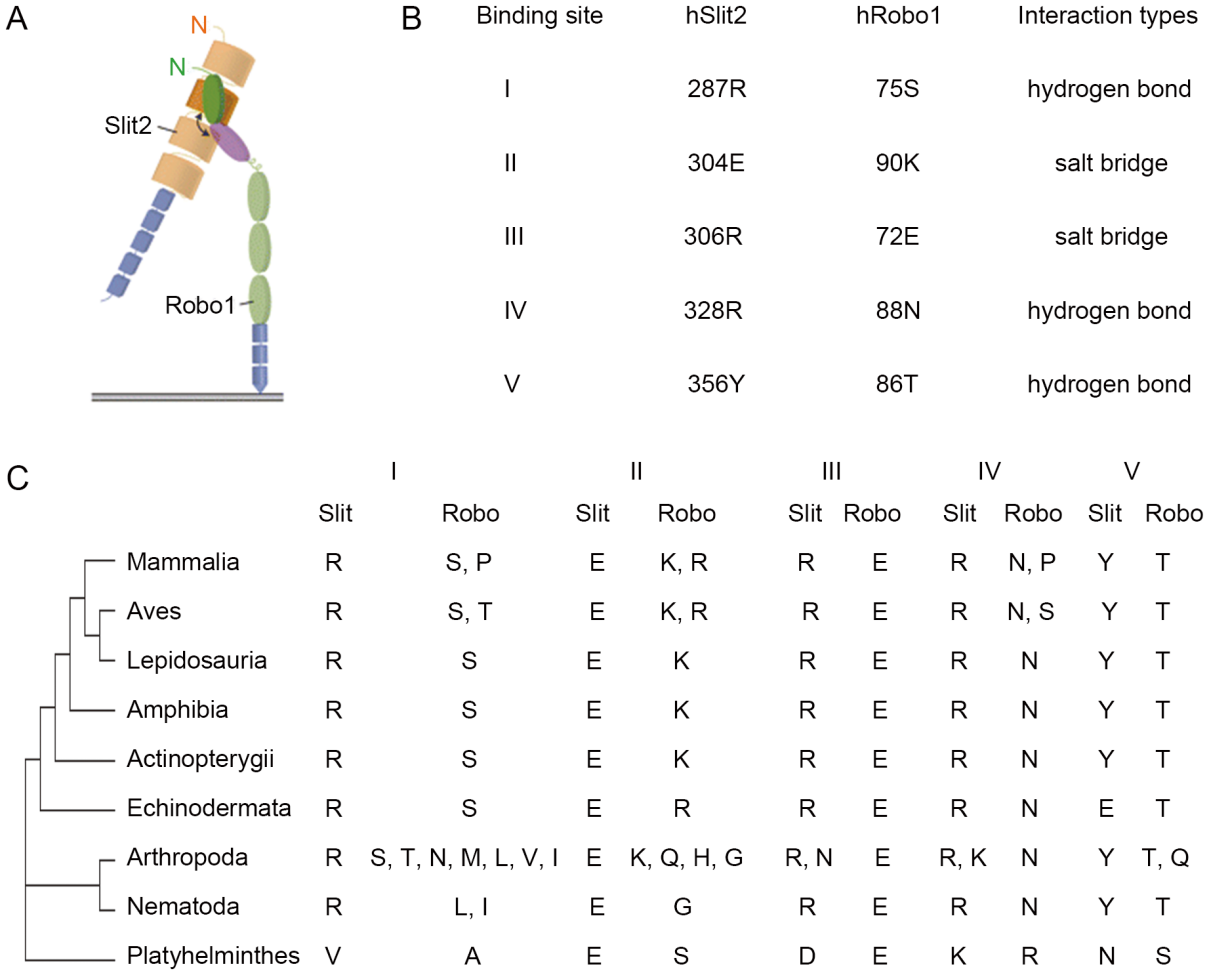
Analysis of interacting amino acids between Slit and Robo. (A) A schematic presentation of the interaction between hSlit2 and hRobo1 through LRR2 domain (orange) bound to Ig1 domain (green) [20]. (B) The binding sites of hSlit2 and hRobo1 identified by SPR spectroscopy [20]. (C) Phylogenetic distribution of interacting amino acids of Slit and Robo.

Detecting correlated changes at specific sites is a commonly used approach for evaluating coevolution of interacting proteins [2]. The interaction of Slit and Robo is mediated through the second LRR domain of Slit and the first Ig domain of Robo (Figure 1A). Five pairs of interacting amino acids between human Slit2 and Robo1 (binding sites I to V) have been identified (Figure 1B) [20]. To understand how these interacting amino acids evolve, we analyzed the sequences of Slit and Robo of various organisms (Figure 1C and Table S5). In Platyhelminthes, the interacting amino acids of Slit and Robo corresponding to human sites I to V were V-A, E-S, D-E, K-R and N-S, whereas site I changed to R-L, R-I, R-S, R-V, R-M, R-N, R-T or R-P; site II changed to E-G, E-K, E-H, E-Q or E-R; site III changed to R-E or N-E; site IV changed to R-N, K-N, R-S or R-P; site V changed to Y-T, Y-Q or E-T during evolution (Figure 1C). Amino acid substitution in Slit appears to accompany the coordinated change in Robo to maintain the Slit-Robo interaction. These results are consistent with the possible coevolution of Slit with Robo.

### Correlated Evolutionary Rate between Slit and Robo

Similarity of phylogenetic trees is also one of the coevolutionary features for interacting proteins, and the distance-based MirrorTree method is an effective approach to assess protein coevolution [3], [4]. The correlations of evolutionary distances between Slit1, 2, 3 and Robo1, 2, 3 from 23 vertebrate species were calculated to test the level of coevolution between Slit and Robo. The correlation coefficient between different protein pairs was shown in Table 1. Robo1 and Robo2 shared similar correlation with three Slit ligands with high average values above 0.9. The highest correlation value 0.991 was identified between Robo1 and Slit2. Robo3 had lower correlation values with three Slits than that of Robo1 and Robo2. Most of the correlation values between Robos and Slits were significantly higher than control groups. These results suggest the coevolution of Slit and Robo in vertebrates.

**Table 1:** Pearson's correlation coefficient of evolutionary distances between Slit and Robo in vertebrates.

	Slit1	Slit2	Slit3	Gapdh
Robo1	0.949**	0.991**	0.961**	0.790
Robo2	0.945**	0.980**	0.961**	0.819
Robo3	0.890**	0.738	0.850**	0.896
Gapdh	0.814	0.757	0.779	1

**the correlation value of Slit-Robo pair is significantly different from controls at 0.01 level.

### Identification of Positively Selected Sites of *slit* and *robo*

**Figure 2:**
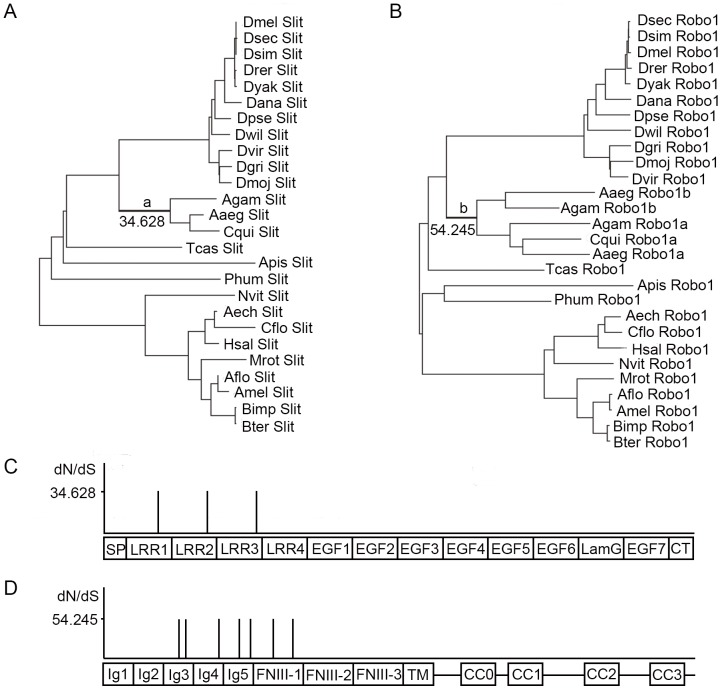
Positive selection of *slit* and *robo1* in insects. (A and B) Phylogenetic trees of *slit* (A) and *robo1* (B) in insects. Both of *slit* and *robo1* under positive selection were detected along the mosquito lineage. The ω values for sites under positive selection along the mosquito lineage were marked. a and b indicate the mosquito lineage. Taxa names are abbreviated with the first letter of the genus and the first three letters of the species. (C and D) Mapping positively selected sites to the Slit (C) and Robo1 (D) proteins.

**Figure 3:**
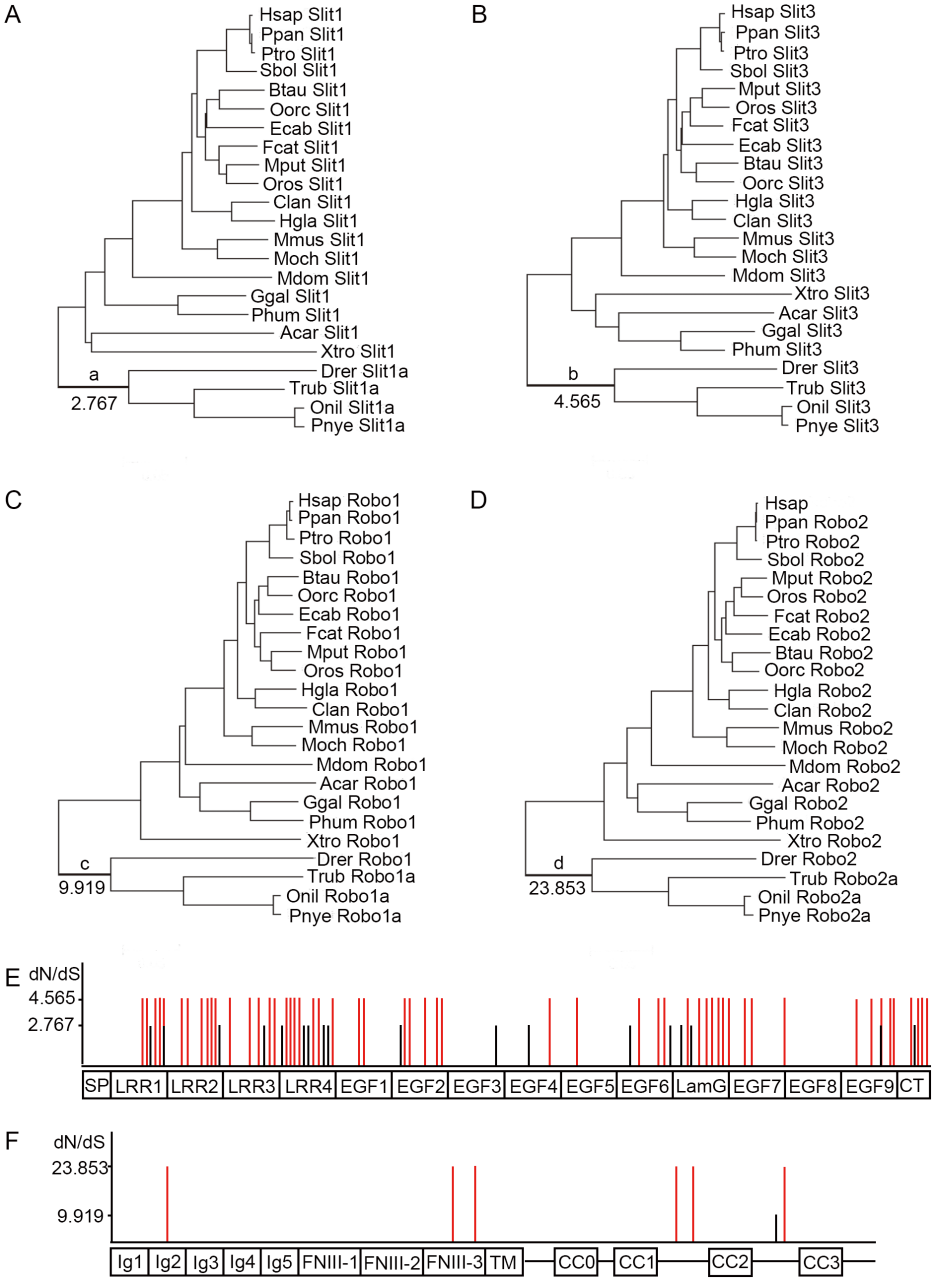
Positive selection of *slit1*, *slit3*, *robo1* and *robo2* in vertebrates. (A–D) Phylogenetic trees of *slit1* (A), *slit3* (B), *robo1* (C) and *robo2* (D). All of these genes under positive selection were detected along the teleost lineages. The ω values of sites under positive selection were marked along the teleost lineage. a, b, c and d indicated the teleost lineage. Taxa names are abbreviated with the first letter of the genus and the first three letters of the species. (E) Mapping positively selected sites identified in *slit1* (black bars) and *slit3* (red bars) to domain structures of Slit. (F) Mapping positively selected sites identified in *robo1* (black bars) and *robo2* (red bars) to domain structures of Robo.

Branch-site model (Model A) of codon evolution was applied to 8 sets of *slit* and *robo* sequences from different species (insects and vertebrates) (Tables S3 and S4). Model A allows a codon site class with ω>1 but only along the foreground branches. A likelihood ratio test (LRT) was used for branch-site models. In the insect datasets, various species were grouped together as the foreground branches (data not shown), but only in the mosquito lineage (including *Aedes aegypti*, *Anopheles gambiae* and *Culex quinquefasciatus*) the LRTs of *slit* and *robo1* were significant correspondingly at the 0.01 level (Figure 2A and 2B, Table S6). This means that *slit* and *robo1* are under strong positive selection at some sites in mosquitoes. Furthermore, 3 positively selected sites of Slit were identified and mapped in its LRR domains (Figure 2C) and 7 positively selected sites of Robo1 were identified and mapped in its Ig and FNIII domains (Figure 2D). In the vertebrate datasets, several different species were grouped together as the foreground branches (data not shown), but only in the teleost lineage the LRTs of *slit1*, *slit3*, *robo1* and *robo2* were significant correspondingly at the 0.01 level (Figure 3 and Table S7). The data suggest that these genes are under positive selection at some sites in the teleost lineage. Moreover, a total of 18 positively selected sites with ω_2_ = 2.767 were identified in Slit1. These sites were located in LRR1, LRR2, LRR3, LRR4, EFG2, EGF3, EGF4, EGF6, LamG, EGF9 and CT domains (Figure 3A and 3E). Up to 54 positively selected sites with ω_2_ = 4.565 were identified in Slit3. The distributions of these positively selected sites are also dispersed: 5 in LRR1, 6 in LRR2, 5 in LRR3, 7 in LRR4, 2 in EGF1, 5 in EGF2, 1 in EGF4, 1 in EGF5, 3 in EGF6, 6 in LamG, 1 in the region between LamG and EGF7, 2 in EGF7, 1 in the region between EGF7 and EGF8, 5 in EGF9, and 4 in CT (Figure 3B and 3E). One site with ω_2_ = 9.919 was identified in Robo1, which resided in the region between CC2 and CC3 (Figure 3C and 3F). Six positively selected sites with ω_2_ = 23.853 were identified in Robo2, which were located in Ig2, FNIII-3, the region between CC1 and CC2, and the region between CC2 and CC3 (Figure 3D and 3F). The LRR, EGF, LamG and CT domains of Slit were all involved in mediating protein-protein interactions. The Ig and FNIII domains of Robo also participated in protein-protein interaction. Both sites under positive selection of *slit* and *robo* that are detected in the mosquito and the teleost lineages support the coevolution of Slit and Robo.

## Discussion

The Slit-Robo couple plays conserved and important roles in the bilaterian central nervous system [13]–[15]. In this study, we used three different methods to detect coevolution of Slit and Robo. Our results provided evidence that *slit* and *robo* have undergone coevolution to maintain the ligand-receptor interaction.

One mechanism of coevolution is the coordinated changes of residues at protein interaction interfaces [25]–[27]. The interaction of Slit and Robo was through five pairs of amino acids in human [20]. During evolution, we found that most of the interacting sites are conserved, while the changes of these interacting amino acids are also identified among several interacting sites which could be classified into three types. The first type of change is that the paired interacting amino acids are conserved in Slit while changed in Robo. In this type, the interaction between basic amino acid and hydrophobic amino acid of R-L changed to R-S which is between basic amino acid and neutral amino acid. Similarly, the interaction between acidic amino acid and neutral amino acid of E-S changed to E-G which is between acidic amino acid and neutral amino acid. This type of changes can not affect the interaction between Slit and Robo. The second type of change is that the paired interacting amino acids are conserved in Robo while changed in Slit. In this type, the interaction between neutral amino acids of Y-T changed to E-T which is between acid amino acid and neutral amino acid, which also has no effect on their interaction. Both Slit and Robo are changed in the third type. In this type, the interaction between hydrophobic amino acids of V-A changed to R-I which is between basic amino acid and hydrophobic amino acid. Although the paired interacting amino acids of N-S changed to Y-T, they are all neutral amino acids. Thus, the interaction between them remains unaffected. Therefore, though the amino acid property of several interacting amino acids changed, their interactions are always conserved. These findings suggest that coordinated changes of interacting amino acids are selected during the coevolution of Slit and Robo. It might be the result of adaptive evolution between Slit and Robo to keep the interaction between them.

In general, interacting proteins evolve at similar rates and showed similar phylogenetic trees [28], [29]. Some factors, such as similar expression patterns, common cellular localization and functioning in a given biochemical pathway, can affect the corresponding proteins in a similar magnitude [30]. We calculated the Pearson's correlation coefficient between the evolutionary rate of Slit and Robo over the whole sequence. Our results suggest a very strong correlation between Slit and Robo. However, Robo3 had lower correlation with three Slit ligands compared with Robo1 and Robo2. It may be due to the weak binding ability of Robo3 with Slits [31], [32]. The high correlation coefficient between Slit and Robo supports the functional association between them and also provides evidence for coevolution.

Recently, some reports showed that the similar pattern of selection for ligands and receptors also represented coevolution, for example, the prolactin-prolactin receptor and the gonadotropin hormones and their receptors [33], [34]. In our study, the branch-site tests for selection were applied to the insect and vertebrate datasets. We tested several different foreground branches for *slit* and *robo*, and the sites under positive selection were only simultaneously detected along the mosquito and teleost lineages. Although the number of *slit* ligand remained constant during invertebrate evolution, the *robo* family underwent independent duplications in insects, with the most family numbers in mosquitoes. Therefore, in mosquitoes the episodic evolution observed for *slit* reflected its adaptation to the presence of multiple *robo* receptors. Due to the fish-specific genome duplication, the interaction between Slit and Robo is more complex within teleosts, which have four Slit ligands and four to seven Robo receptors. In teleosts, *slit1* and *slit3* subjected positive selection, and the same happened in *robo1* and *robo2*. The similar pattern of selection for *slit* and *robo* further supports the coevolution of the two genes. One important role for the Slit-Robo couple is midline repulsion, which is well-conserved in the Bilateria. The positive selection acting on *slit* and *robo* was probably associated with their functional adaptation.

Taken toghter, we analyzed the coevolutionary characteristics of Slit ligand and Robo receptor from many aspects. This study will provide a theoretical background for the evolution of axon guidance molecules.

## Supporting Information

Table S1 **The accession numbers of sequences used in analysis of interacting amino acid changes.**
(XLS)

Table S2 **The accession numbers of sequences used in correlated evolution rate analysis.**
(XLS)

Table S3 **The accession numbers of insect sequences used in PAML selection analysis.**
(XLS)

Table S4 **The accession numbers of vertebrate sequences used in PAML selection analysis.**
(XLS)

Table S5 **Analysis of changes in the interacting sites between Slit and Robo.**
(XLS)

Table S6 **Parameter estimates of branch-site models for** ***slit*** **and** ***robo1*** **in insects.**
(XLS)

Table S7 **Parameter estimates of branch-site models for** ***slit1*** **,** ***slit3*** **,** ***robo1*** **and** ***robo2*** **in vertebrates.**
(XLS)
