# Correction: Coevolution of Axon Guidance Molecule Slit and Its Receptor Robo

**DOI:** 10.1371/journal.pone.0110582

**Published:** 2014-10-06

**Authors:** The PLOS ONE Staff

There are errors in the author affiliations. The affiliations should appear as shown here:

Qi Yu^1^, Xiao-Tong Li^1^, Xiao Zhao^1^, Xun-Li Liu^1^, Kazuho Ikeo^2^, Takashi Gojobori^2,3^, Qing-Xin Liu^1^

1 Laboratory of Developmental Genetics, Shandong Agricultural University, Tai’an, Shandong, China, 2 Center for Information Biology and DNA Data Bank of Japan, National Institute of Genetics, Mishima, Shizuoka, Japan, 3 Computational Bioscience Research Center, Biological and Environmental Sciences and Engineering, KAUST (King Abdullah University of Science and Technology), Thuwal, Saudi Arabia
